# Occupationally Relevant Zinc‐ and Copper‐Containing Metal Fumes Inhibit Human THP‐1 Macrophage TNF and IL‐6 Responses to Bacterial Stimuli

**DOI:** 10.1002/gch2.202400302

**Published:** 2024-12-31

**Authors:** Jan Steffens, Sabrina Michael, Katharina Kuth, Henner Hollert, Miriam Du Marchie Sarvaas, Andrijana Nesic, Thomas Kraus, Ralf Baumann

**Affiliations:** ^1^ Institute for Translational Medicine (ITM) Medical School Hamburg (MSH) 20457 Hamburg Germany; ^2^ Institute for Occupational, Social and Environmental Medicine Medical Faculty University Hospital RWTH Aachen University 52074 Aachen Germany; ^3^ Institute of Hygiene and Environmental Medicine Medical Faculty University Hospital RWTH Aachen University 52074 Aachen Germany; ^4^ Department Evolutionary Ecology and Environmental Toxicology Institute of Ecology, Evolution and Diversity Faculty Biological Sciences Goethe University Frankfurt 60438 Frankfurt Germany

**Keywords:** CuO/ZnO particles, immunosuppressive effects on macrophages, metal (nano)‐particle exposure, welding and metal fumes, workplace safety and new approach methodologies (NAM)

## Abstract

Metal workers have an increased risk of severe lobar pneumonia due to exposure to metal fume particles, which lead to recent pneumococcal vaccination recommendations. To investigate the effects of metal fume‐derived zinc oxide (ZnO) and copper oxide (CuO) particles on airway immune responses, human THP‐1‐derived macrophages are exposed in vitro to the bacterial pathogen‐associated molecular patterns (PAMPs) lipopolysaccharide (LPS), lipoteichoic Acid (LTA), or peptidoglycan (PGN), together with particle suspensions. Particles are generated through metal inert gas (MIG) soldering. Spectrometric and microscopic analysis confirms CuO and ZnO as main components. Macrophage *IL‐6* and *TNF* mRNAs are quantified by qPCR and secreted protein levels by electrochemiluminescent multi‐spot assay. A dose‐dependent increase in macrophage TNF and IL‐6 mRNA (4 h) and protein (24 h) levels following exposure to PAMPs is significantly inhibited by 2 µg mL^−1^ CuO/ZnO particles (*n* = 5). Additionally, CuO/ZnO particles significantly inhibit TNF protein expression in unstimulated macrophages, while IL‐6 protein levels are unaffected (*n* = 5). The presented in vitro immunotoxicity approach may extend existing new approach methodology (NAM) elements for chemical risk assessment and possibly exposure limit evaluation refinements. These findings implicate that CuO/ZnO particles suppress macrophage proinflammatory responses to PAMPs, potentially compromising lung immunity, underlining current vaccine recommendations and efforts for preventive occupational health guidelines.

## Graphical abstract

Metal workers have an increased risk of severe pneumonia due to metal fume exposure. In this study, zinc oxide (ZnO) and copper oxide (CuO) particles are found to inhibit specific anti‐bacterial macrophage responses, which may substantially contribute to impaired lung immunity. These findings underline the importance of preventive occupational health guidelines and current pneumococcal vaccine recommendations for long‐term metal workers.

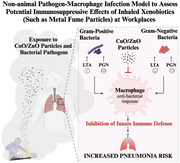

## 1 Introduction

Long‐term exposure (for several years) of metal workers or welders to metal fumes is associated with several health risks,^[^ ^1^ ^]^ including cardiovascular diseases,^[^ ^2^, ^3^ ^]^ as well as airway diseases such as pulmonary carcinogenicity,^[^ ^4^ ^]^ severe chronic obstructive pulmonary disease (COPD),^[^ ^5^, ^6^ ^]^ respiratory inflammation,^[^ ^7^, ^8^ ^]^ increased airway responsiveness,^[^ ^9^ ^]^ bronchitis,^[^ ^10^, ^11^ ^]^ rhinitis and welding‐related asthma,^[^ ^12^ ^]^ and fibrosis.^[^ ^13^ ^]^ In addition, metal fume exposure is reported to exacerbate the severity, frequency, and duration of respiratory tract infections in welding laborers, which leads to a higher risk of lobar pneumonia.^[^ ^14^, ^15^, ^16^, ^17^, ^18^, ^19^ ^]^ The severity of this issue is underscored by an increased mortality rate among metal workers, particularly welders, due to invasive pneumococcal disease (IPD).^[^ ^20^, ^21^, ^22^ ^]^

Pneumococcal disease is caused by several bacteria, such as the Gram‐positive bacteria *Staphylococcal aureus* and particularly *Streptococcus pneumoniae*. Indeed, *Streptococcus pneumoniae* (with currently over 90 known distinct serotypes) is, to a large extent, responsible for community‐acquired pneumonia,^[^ ^23^, ^24^ ^]^ mortality during flu outbreaks,^[^ ^25^, ^26^ ^]^ and admission of severe community‐acquired pneumonia patients into intensive care units.^[^ ^27^, ^28^ ^]^ Pneumococcal vaccines that protect against clinically relevant serotypes of *Streptococcus pneumoniae* are recommended in the UK^[^ ^29^ ^]^ and Germany^[^ ^28^, ^30^ ^]^ for adults aged 65 and above and people with certain health conditions (e.g., immunodeficiency, chronic diseases of the heart or respiratory organs).

As *Streptococcus pneumoniae* is also the main cause of severe lung pneumonia of long‐term metal workers,^[^ ^17^, ^19^, ^21^, ^31^ ^]^ the Health and Safety Executive (HSE) in the United Kingdom^[^ ^27^ ^]^ and the German Standing Committee on Vaccination (STIKO) of the Robert Koch Institute (RKI) both strongly recommend pneumococcal vaccines for metal workers since 2014 and 2016, respectively.^[^ ^28^, ^29^, ^32^ ^]^ Taken together, the metal fumes seem to dampen the human lung immune responses to bacterial infection.

The different metals used in metal construction operations, such as iron (Fe), chromium (Cr), nickel (Ni), zinc (Zn), copper (Cu), and manganese (Mn), cause different health implications. In the present study, the focus lays on the immunotoxicological effects of Zn and Cu for several reasons: ZnO nanostructure research is ongoing as ZnO nanostructures are advantageous for many applications in sensing, photocatalysis, functional textiles, and cosmetic industries.^[^ ^33^ ^]^ Furthermore, the role of Zn and Cu in modern joining technology, particularly in the automotive industry, is increasing,^[^ ^34^ ^]^ and therefore, metal workers are more frequently exposed. Short‐term exposure (for several hours or days) to Zn and ZnO fumes, and in some reports also to Cu and CuO fumes, cause metal fume fever (MFF), a medical condition characterized by fever, headache, and respiratory symptoms.^[^ ^35^, ^36^, ^37^, ^38^, ^39^ ^]^ In previous studies, we found in healthy individuals significant systemic increases of acute phase proteins after a short‐term (6 h) exposure to occupationally relevant welding fumes containing ZnO and CuO at a combined dose of 2.5 mg m^−3^,^[^ ^40^, ^41^ ^]^ but not after inhalation of welding fumes containing either iron, nickel, chromium^[^ ^42^ ^]^ or aluminum.^[^ ^40^ ^]^ Likewise, we found comparable systemic effects for the inhalation of metal fumes containing “only Zn” (1.2 mg m^−3^) or “only Cu” (0.34 mg m^−3^).^[^ ^43^, ^44^ ^]^ The systemic elevation of acute phase responses was confirmed by another research group, which exposed healthy individuals for four consecutive days to either 0.5, 1, or 2 mg m^−3^ pure nano‐sized ZnO particle concentrations, with significant effects already at 1.0 mg m^−3^. As this concentration is well below the occupational exposure limit for ZnO in many countries, this group has recommended to reassess the exposure limit value for ZnO at workplaces.^[^ ^45^, ^46^ ^]^ Other groups showed for workers in field studies at workplaces significant systemic increases of CRP and/or IL‐6 after short‐term exposures to welding fume (not explicitly Zn or Cu) and/or other occupational air pollutants.^[^ ^47^, ^48^ ^]^ In a further study, we showed that short‐term exposure to ZnO‐ and CuO‐containing metal fumes caused early nasal increases of acute phase proteins,^[^ ^49^ ^]^ thereby confirming that the inflammation starts in the airways. Moreover, in vitro, comparative toxicity studies using 24 manufactured nanoparticles showed that Cu‐ and Zn‐based nanomaterials were the most toxic nanoparticles for human macrophages.^[^ ^50^ ^]^ Specifically, ZnO nanoparticles have been linked to lysosomal destabilization, thereby disturbing macrophage functionality.^[^ ^13^ ^]^ Taken together, ZnO and CuO affect macrophages, potentially compromising their responses to bacterial lung infections.

To enable a mechanistic investigation of the potentially inhibitory effects of metal fume exposure on lung immune responses to bacterial infection, in the current study, we used a simplified in vitro model: Human macrophages, as important lung immune cell type,^[^ ^51^, ^52^, ^53^, ^54^ ^]^ were exposed to three different pathogen‐associated molecular patterns (PAMPs) (lipopolysaccharide (LPS), lipoteichoic acid (LTA) or peptidoglycan (PGN)) to simulate an infection scenario with gram‐positive or ‐negative bacteria, in the presence or absence of metal fume‐derived ZnO and CuO particle suspensions.

## 2 Experimental Section

### 2.1 Metal Fume Particle Generation

To generate the CuO/ZnO particles, metal inert gas (MIG) soldering was manually performed by a skilled welder within the confines of a funnel‐shaped fume hood. Utilizing a hot‐dip zinc‐coated steel sheet (EN 10346: DX51D+Z275) as the base material, the filler metal comprised 96% Cu, 1% Mn, and 3% silicon (Si) (CuSi3Mn;) (Bercoweld S3, Bedra, Germany). Argon served as the shielding gas during this procedure. The resulting fumes were captured within the fume hood and collected as metal particle dust, following established methodologies.^[^ ^40^ ^]^

### 2.2 Metal Fume Particle Characterization by Electron Microscopy

A suspension of CuO/ZnO particles was prepared using ethanol and ultra‐pure water as solvents. 100 and 2 µg mL^−1^ of CuO/ZnO of the metal fume particles were resuspended in ethanol and ultrapure water, respectively. Subsequently, the CuO/ZnO particle suspensions underwent sonication in an ultrasonic bath for 30 minutes, followed by storage in a freezer at ‐20 °C. Prior to application, the suspensions were thoroughly mixed by vortexing. The prepared suspension was then applied onto a sample grid and evaporated under a fume hood to obtain a thin layer of particles. Scanning electron microscope (SEM) images of the particles were acquired using a field emission scanning electron microscopy (SEM; Zeiss Gemini 982 SEM, Zeiss, Oberkochen, Germany). Elemental analysis of the welding fume particles was conducted using energy‐dispersive X‐ray spectroscopy (EDX) with an Oxford ISIS EDS detector (Wiesbaden, Germany).

### 2.3 Metal Fume Particle Characterization by Inductively Coupled Plasma Mass Spectrometry (ICP‐MS)

Initially, 50 mg of the metal fume particles were added to an aqueous solution within a decomposition vessel. This solution consisted of 1 mL of rhodium‐ICP standard solution, 7 mL of hydrogen peroxide, and 5 mL of aqua regia (75% (v/v) HCl, 25% (v/v) HNO_3_). Subsequently, microwave digestion was performed for 1 h. Following digestion, the chilled particle suspension was diluted to a ratio of 1:10 using aqua bidest and then filtered. To the resulting solution, 20 µL of nitric acid and 100 µL of rhodium‐ICP were added to 9.88 mL of the filtered solution. Analysis of these solutions was conducted using the ICP Mass Spectrometer ELAN DRC II (PerkinElmer Inc., Waltham, Massachusetts, USA). Quantification was achieved by comparing the results to measured standards, including 10% (v/v) ICP‐multi‐element standard solution (ME) VI and 10% (v/v) Sn‐ICP Standard 1%. Based on the determined values, the aqueous‐available fraction of the metals was calculated.

### 2.4 Optimal Concentration of Cu/Zn Metal Fumes for In Vitro Study of Cellular Responses

The concentration of 2 µg mL^−1^ did not result in significant macrophage cell death. To assess the adequacy of this in vitro concentration for accurately simulating actual occupational conditions, the methodology was applied proposed by McCarrick et al. to compare in vitro and in vivo exposure concentrations.^[^ ^55^ ^]^ The concentration of 2 µg mL^−1^ of CuO/ZnO particles, equivalating to 1.1 µg cm^−^^2^ (calculation based on: 24‐well plate; 1 mL medium volume, 1.82 cm^2^ growth surface area), falls within their investigated range considering MPPD modeling (deposition, clearance, retention) and is relatively low. McCarrick defines short‐term exposure as 6 h to 1 week and long‐term exposure as 1 year to 45 years. This concentration exceeds the alveolar retention after 1 week of exposure to the occupational exposure limit (OEL) of 5 mg m^−^^3^ (0.083 µg cm^−^^2^) but is lower than the retention after 1 year (2.85 µg cm^−^^2^). It also corresponds to the tracheobronchial retention of 0.89 µg cm^−^^2^ after 6 h at the OEL, indicating relevance for short‐term scenarios. Furthermore, 1.1 µg cm^−^^2^ exceeds retention levels observed in both tracheobronchial (0.023 µg cm^−2^) and alveolar regions (0.16 µg cm^−2^) after 1 week of exposure to low‐end concentrations (0.05 mg m^−^^3^). This suggests its use for investigating both short‐ and long‐term exposure effects, as it represents a potential concentration in different lung regions based on exposure duration and concentration. While 1.1 µg cm^−^^2^ equals the tracheobronchial retention level of 1.15 µg cm^−^^2^ long‐term, it remains below the alveolar retention of 2.85 µg cm^−^^2^ after one year. It is essential to note that in vitro concentrations often represent a bolus dose, while in vivo deposition typically occurs over an extended exposure period, such as several hours. Additionally, in vitro systems frequently require higher concentrations to produce observable effects due to their relative simplicity compared to in vivo conditions.^[^ ^55^ ^]^

### 2.5 Cell Culture Conditions and Exposure Experiments

Monocytic THP‐1 cells were cultured in RPMI 1640 medium (Gibco, Thermo Fisher Scientific Inc., Waltham, Massachusetts, USA), supplemented with 10% low‐endotoxin fetal bovine serum (FBS) Sera Plus (PAN Biotech, Aidenbach, Germany), 2 mm L‐glutamine, 100 U mL^−1^ penicillin, and 100 µg mL^−1^ streptomycin (Gibco, Thermo Fisher Scientific Inc., Waltham, Massachusetts, USA), within a 5% CO_2_ humidified atmosphere at 37 °C. To induce monocytic differentiation, cells were exposed to 100 nm phorbol 12‐myristate 13‐acetate (PMA; Merck KGaA, Darmstadt, Germany) at a concentration of 5.5 × 10^5^ cells mL^−1^ for 48 h. Following differentiation to macrophages, the PMA‐containing medium was replaced with assay medium comprising 1% FBS and 1% penicillin/streptomycin. After an additional 24 h incubation, the adherent macrophages were subjected to various cell culture media suspensions (as delineated in **Table** **1**) under a 5% CO_2_‐humidified atmosphere at 37 °C.

**Table 1:** Exposure substances and conditions overview. The following table summarizes the concentrations of the exposure substances used and their respective exposure conditions. The symbols “+” and “++” indicate lower and higher concentrations, respectively. The abbreviations used are as follows: LPS, LTA, PGN, and CuO/ZnO (Cu/Zn‐containing metal fume particles).

	Substance [ng mL^−1^]						
	LPS		LTA		PGN		CuO/ZnO
Concentration	2	20	100	1000	1000	3162	2000
Exposure level	+	++	+	++	+	++	+

To assess macrophage responses to bacterial stimuli, PAMPs were employed instead of live bacteria. LPS from *Escherichia coli* O111:B4 (Merck KGaA, Darmstadt, Germany) was used to represent Gram‐negative bacteria, while LTA (InvivoGen, Toulouse, France) represented Gram‐positive bacteria, and PGN (InvivoGen, Toulouse, France) served as representative of Gram‐positive and Gram‐negative bacteria, respectively. Both LTA and PGN were obtained from *Staphylococcus aureus* because of their commercial availability and low endotoxin levels. *Staphylococcus aureus*, similar to *Streptococcus pneumoniae*, is a well‐known cause of community‐acquired pneumonia and can be preceded by influenza infection, thus promoting the emergence of low‐virulent strains.^[^ ^56^ ^]^ Moreover, the emergence of methicillin‐resistant *Staphylococcus aureus* (MRSA) underlines the clinical importance of this bacterial strain.^[^ ^57^ ^]^ The chemical structure and properties of LTA from *Staphylococcus aureus* and *Streptococcus pneumoniae* are identical, and their signaling effects were reported to be similar.^[^ ^58^, ^59^ ^]^

For RNA purification, cells were harvested using QIAzol (Qiagen GmbH, Hilden, Germany) lysis buffer at 0 and 4 h. The resulting homogenized samples were then transferred to clean tubes pre‐cooled at 4 °C and stored at ‐80 °C until further processing.

### 2.6 Cell Viability and Toxicological Assessment

The toxicity of the exposure substances on THP‐1 macrophages was evaluated using the 3‐(4,5‐dimethylthiazolyl‐2)‐2,5‐diphenyltetrazolium bromide (MTT) cell proliferation assay following the manufacturer's instructions (MTT# 30–101K, ATCC Rockville, MD, USA). THP‐1 cells were seeded in triplicate at a density of 10^3^ cells mL^−1^ and incubated for 24 h at 37 °C in a 5% CO_2_ incubator. Subsequently, the testing reagents were added: 2, 4, 6, 8, and 16 µg mL^−1^ CuO/ZnO particles, LPS at 2 ng mL^−1^ (+ 2 µg mL^−1^ CuO/ZnO particles), LTA at 100 ng mL^−1^ (+ 2 µg mL^−1^ CuO/ZnO particles), PGN at 1000 ng mL^−1^ (+ 2 µg mL^−1^ CuO/ZnO particles). RPMI 1640 medium served as a control to establish the baseline for absorbance readings. After incubation of 24 h, 10 µL of the MTT reagent was added and incubated for an additional 4 h, followed by the addition of 100 µL of the detergent reagent. Following an overnight incubation period in the dark, absorbance readings were obtained at 570/670 nm using an automatic ELISA plate reader SpectraMax iD3 (Molecular Devices, San Jose, USA). The optical density (OD) was measured for cultures exposed to both exposure and non‐exposure media, as detailed in Table 1.

### 2.7 Cell Sample Preparation and Gene Expression Analysis

The total RNA of the cells was extracted using a phenol‐based lysis buffer, QIAzol, as part of the miRNeasy Mini Kit (Qiagen GmbH, Hilden, Germany), following the manufacturer's protocol. Subsequently, 1 µg of total RNA was reverse‐transcribed using the Applied Biosystems High‐Capacity cDNA Reverse Transcription Kit (Thermo Fisher Scientific Inc., Waltham, Massachusetts, USA). Quantitative real‐time Polymerase Chain Reaction (qPCR) was conducted using 5 µL of cDNA in 20 µL reaction volumes (in duplicates) with the SensiFAST SYBR Lo‐ROX Kit (Bioline GmbH, Luckenwalde, Germany) on the StepOnePlus Real‐Time PCR System (Thermo Fisher Scientific Inc., Waltham, Massachusetts, USA). Primer pairs for *IL‐6*, *TNF*, and Glyceraldehyde 3‐phosphate dehydrogenase (*GAPDH*) genes are detailed in **Table** **2**. The qPCR protocol consisted of an initial denaturation step at 95 °C for 3 min, followed by 40 cycles of denaturation at 95 °C for 5 s, annealing at 62 °C for 10 s, and extension at 72 °C for 20 s. *GAPDH* served as the reference gene for data normalization.

**Table 2:** Sequences of the mRNA qPCR primer pairs. The mRNA primer pairs chosen for the RT‐qPCR analysis are shown below. The applied concentration of each primer was 0.5 µm for qPCR measurements. The abbreviations used in the table are as follows: F ((forward) – [5′‐primer sequence‐3′]), R ((reverse) – [5′‐primer sequence‐3′]).

Gene	Accession	Sequence (5´→ 3´)	References
*TNF*	NM_000594.4	F – CTGCTGCACTTTGGAGTGAT	[60]
		R – AGATGATCTGACTGCCTGGG	
*IL‐6*	NM_000600.3	F – TCGAGCCCACCGGGAACGAA	[61]
		R – GTGGCTGTCTGTGTGGGGCG	
*GAPDH*	NM_002046.3	F – GGAGAAGGCTGGGGCTCAT	[62]
		R – TGATGGCATGGACTGTGGTC	

### 2.8 Multispot Electrochemiluminescence Immunoassay (ECLIA) for TNF and IL‐6

For quantifying TNF and IL‐6 protein levels, supernatants were collected 24 h post‐treatment and stored immediately at ‐80 °C. The supernatants were thawed only once right before analysis to ensure accuracy. The detection of TNF and IL‐6 was performed using the Meso Scale Discovery V‐PLEX following the provided manufacturer's instructions. Measurements were conducted on the MESO QuickPlex SQ 120 instrument, using DISCOVERY WORKBENCH 4.0 software for analysis. The reported protein levels of IL‐6 and TNF are the average from three separate experiments, presented with their standard error of the mean.

### 2.9 Statistical Analysis

Experimental data were subjected to statistical analysis using GraphPad Prism software version 10 (GraphPad Software, Inc., San Diego, USA). A two‐tailed *P*‐value of less than 0.05 was considered statistically significant. Data obtained from RT‐qPCR was expressed as relative quantification (RQ; 2^−∆∆Ct^) of the mean, with RQ_min_ and RQ_max_ representing the range of possible RQ values based on the standard error of the mean (2^(‐∆∆Ct) ± standard error of the mean^) for each sample. The standard error of the mean of ΔCT was determined by five independent experiments (*n* = 5). Statistical analysis of ‐log2(fold change) was conducted by using the Shapiro‐Wilk test, followed by ratio‐paired t‐test analysis.

Protein concentration in the supernatant was determined through three independent experiments (*n* = 3). The statistical analysis was conducted using the Shapiro‐Wilk test, followed by ratio‐paired t‐test analysis. Results were presented as mean ± standard error of the mean.

Cell viability results were obtained from three independent experiments (*n* = 3). Statistical testing was carried out using the Shapiro‐Wilk test, followed by the ratio‐paired t‐test analysis. Results were presented as mean ± standard error of the mean.

For concentrations of CuO/ZnO particles demonstrating a minimum of two viabilities lower than 50% of the control condition, the IC_50_ value (inhibitory concentration 50; concentration causing 50% cell mortality) was determined. The X‐values underwent a logarithmic transformation, and non‐linear regression analysis was conducted using a sigmoidal dose‐response model with variable slope, setting bottom and top constraints at 0 and 100, respectively. The values were expressed as mean ± 95% confidence intervals (CI).

## 3 Results and Discussion

### 3.1 Physicochemical Analysis of the Metal Fume Particles

**Figure 1:**
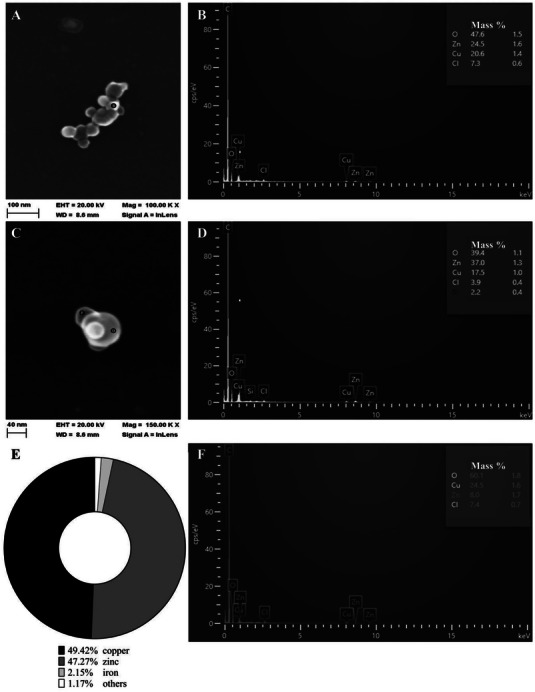
Physicochemical analysis of the metal fume particles. A,C) SEM micrographs of metal fume particles in aqueous solution. Metal fume particles were suspended in an aqueous solution for SEM analysis. The particles are shown at A) 100 000× or C) 150 000× magnification. The images reveal spherical particle shapes. A,C) Contain three black circles with B,D, or F) white letters to denote measurement sites of the respective EDX data. B,D,F) EDX spectra of metal fume particles. EDX mapping analysis illustrates the spatial distribution of elements within a particle cluster, confirming the presence of oxygen, Zn, and Cu as significant components. E) ICP‐MS analysis of metal fume particles. The metal fume predominantly consists of 49.42% Cu and 47.27% Zn, with minor contributions from iron (2.15%) and residual components (1.17%), indicating a complex chemical composition.

The particle size distribution of the occupationally relevant metal fume was previously reported to be in the range between 40 and 250 nm, with the modal size at 124 nm and 33% of the particles being smaller than 100 nm, representing ultrafine metal particles.^[^ ^40^ ^]^ The metal fume particle suspension was analyzed using ICP‐MS, EDX, and SEM (**Figure** **1**). ICP‐MS analysis showed that the metal fume particles consisted of 49.42% Cu, 47.27% Zn, 2.15% Fe, and 1.17% residual components (Figure 1E). EDX analysis confirmed CuO and ZnO as the primary components of the single particles and particle clusters (Figure 1B,D,F), and we therefore refer to these welding fume particles as CuO/ZnO particles. In addition, SEM examination of the welding fume particles in suspension identified spherical particle shapes (Figure 1A,C). This shape is characteristic of metals undergoing first evaporation at high temperatures, followed by condensation, during metal joining processes.^[^ ^63^, ^64^ ^]^

The generation of metal fume particles during metal‐joining processes results in significant compositional variability due to factors such as filler material, base material, flux components, shielding gas, and process parameters.^[^ ^11^ ^]^ This variability explains the observed differences between the analyses of the entire sample (ICP‐MS) (Figure 1E) and the individual particle sites (EDX) (Figure 1B,D,F). ICP‐MS analysis of the metal fume particles represents the average particle composition of the entire sample, which revealed similar amounts of Cu and Zn (Figure 1E). In contrast, EDX analyses (Figure 1B,D,F) taken from concrete measurement sites (the locations are depicted in Figure 1A,C) showed varying Cu‐to‐Zn ratios and thus a heterogeneity of the individual metal fume particles. As stated, these disparities stem from the inherently stochastic nature of particle formation during metal‐joining processes. The presented findings underscore the importance of a dual analysis approach using ICP‐MS and EDX for fully characterizing metal fume particles.^[^ ^65^, ^66^ ^]^

### 3.2 Cytotoxicity Testing of CuO/ZnO Particles and/or PAMPs Exposure on THP‐1 Macrophages

**Figure 2:**
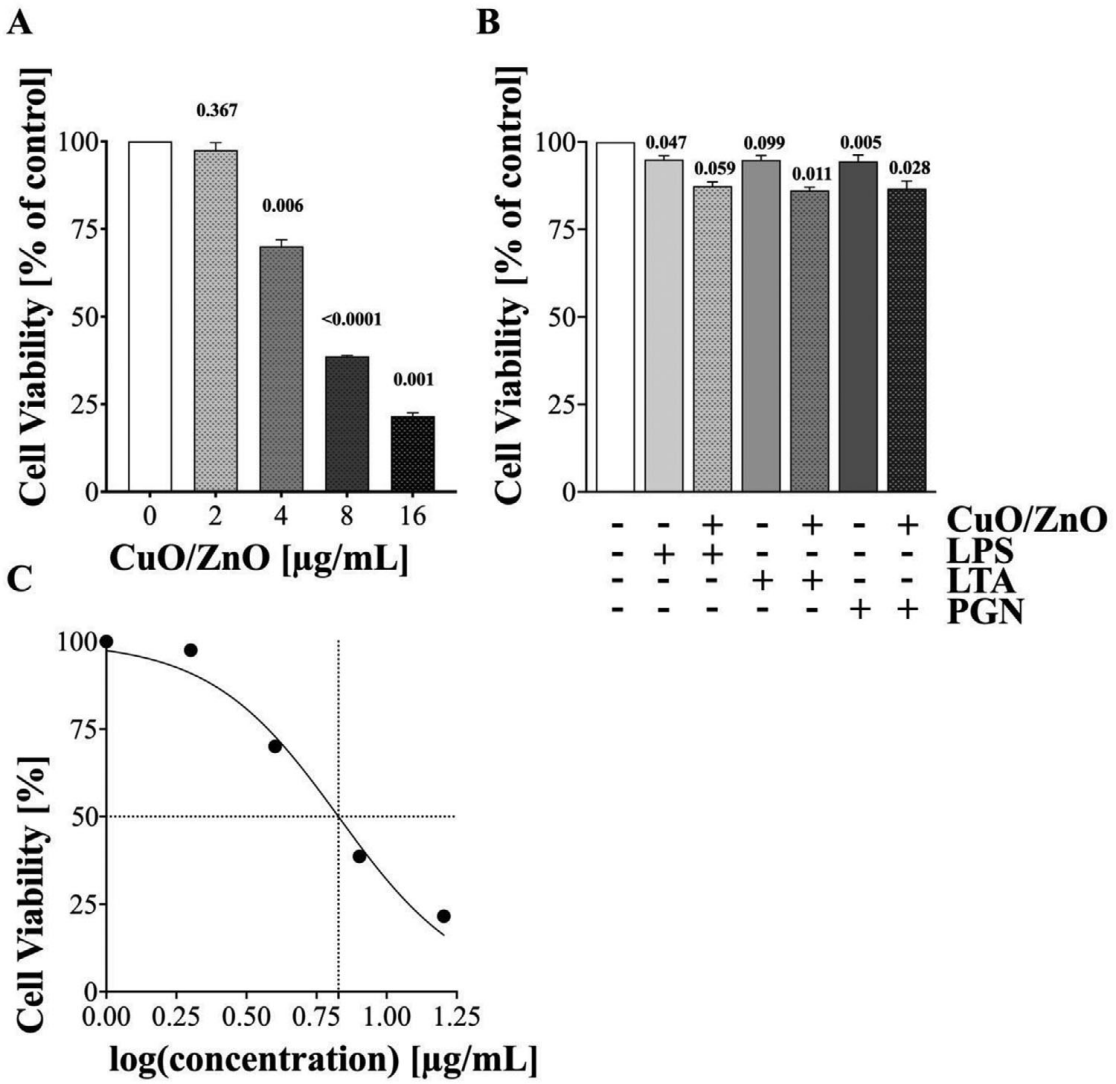
Evaluation of the cytotoxicity of CuO/ZnO particles and/or PAMPs on THP‐1 macrophages. A) Dose‐dependent cytotoxicity of CuO/ZnO particles. THP‐1 macrophages were exposed to increasing concentrations of CuO/ZnO particles (2, 4, 8, and 16 µg mL^−1^) for 24 h. The viability, determined by the MTT assay, decreased dose‐dependently (97.52 ± 3%, 70.01 ± 1.6%, 38.64 ± 0.3%, and 21.58 ± 0.8%, respectively). B) Immunotoxic effects of CuO/ZnO particles on PAMP‐exposed THP‐1 macrophages. At exposure with PAMPs (LPS at 2 ng mL^−1^, LTA at 100 ng mL^−1^, or PGN at 1000 ng mL^−1^) alone, high cell viabilities were detected at 24 h (ranging from 91.95 ± 0.9% to 94.43 ± 1.0%), and co‐exposure with 2 µg mL^−1^ CuO/ZnO particles resulted in decreased viabilities (87.36 ± 1.0% for LPS‐, 86.15 ± 0.8. 8% for LTA‐, and 86.7 ± 1.8% for PGN‐activated cells). C) The IC_50_ concentration for the macrophage cytotoxicity of CuO/ZnO particle exposure. This value was determined to be 6.728 µg mL^−1^ at 24 h after exposure using the MTT cell proliferation test.

To determine the toxicity of these CuO/ZnO particles on human THP‐1 macrophages, different concentrations of the particles were tested by the MTT assay using three independent experiments (*n* = 3). The MTT assay showed a dose‐dependent decrease in cell viability after a 24 h exposure to increasing concentrations of CuO/ZnO particles. Specifically, concentrations of 4, 8, and 16 µg mL^−1^ resulted in significant decreases in the cell viability, down to 70.01 ± 1.6%, 38.64 ± 0.3%, and 21.58 ± 0.8%, respectively, compared to the nonexposed control at 24 h (**Figure** **2**A). The concentration of 2 µg mL^−1^ did not result in significant cell death (97.52 ± 3% viability). The IC_50_ value, as the concentration at which 50% of cells exhibited toxicological effects, was determined to be 6.728 µg mL^−1^ (Figure 2C).

A cellular pathogen‐host infection model was simulated by inducing pro‐inflammatory responses in the THP‐1‐derived macrophages using the PAMPs LPS, LTA, and PGN. When THP‐1 macrophages were treated with PAMPs (LPS, LTA, or PGN) alone, the cell viabilities remained relatively high, ranging from 91.95 ± 0.9% to 94.43 ± 1.0% (Figure 2B). However, co‐exposure to 2 µg mL^−1^ CuO/ZnO particles alongside PAMPs for 24 h resulted in decreased cell viabilities: 87.36 ± 1.0% for LPS‐activated cells, 86.15 ± 0.8.8% for LTA‐activated cells, and 86.7 ± 1.8% for PGN‐activated cells.

### 3.3 Inflammatory Effects of PAMPs on THP‐1 Macrophages in the Absence or Presence of CuO/ZnO Particles

**Figure 3:**
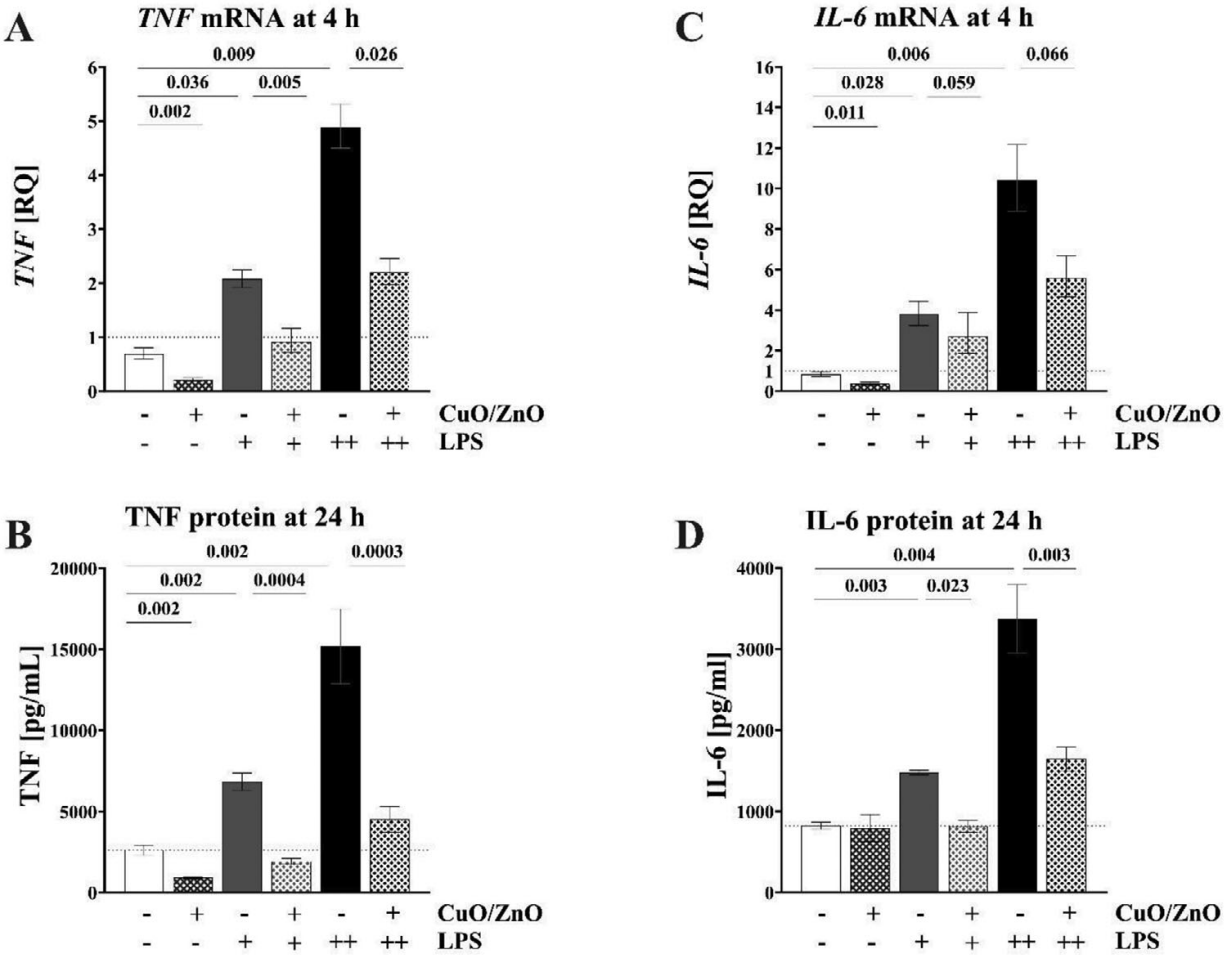
Effects of CuO/ZnO particles on THP‐1 macrophages. A) *TNF* mRNA expression after 4 h exposure to 2 µg mL^−1^ CuO/ZnO particles, measured using RT‐qPCR. B) TNF protein levels after 24 h of exposure to 2 µg mL^−1^ CuO/ZnO particles, measured using ECLIA. C) *IL‐6* mRNA expression after 4 h of exposure to 2 µg mL^−1^ CuO/ZnO particles, measured using RT‐qPCR. D) IL‐6 protein levels after 24 h of exposure to 2 µg mL^−1^ CuO/ZnO particles, measured using ECLIA. LPS concentrations used: + for 2 ng mL^−1^ and ++ for 20 ng mL^−1^.

**Figure 4:**
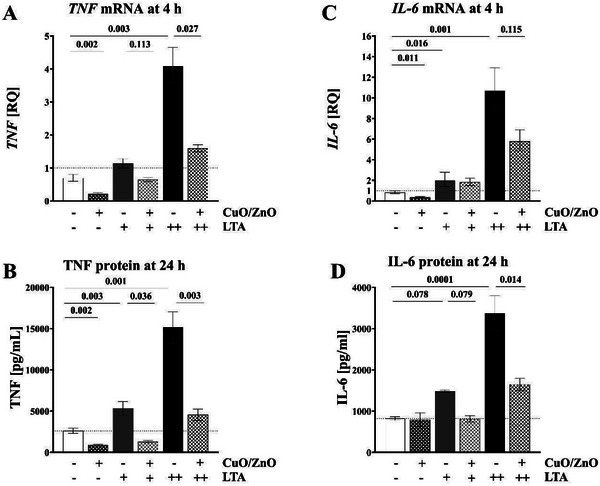
Effects of CuO/ZnO particles on THP‐1 macrophages. A) *TNF* mRNA expression after 4 h of exposure to 2 µg mL^−1^ CuO/ZnO particles, measured using RT‐qPCR. B) TNF protein levels after 24 h of exposure to 2 µg mL^−1^ CuO/ZnO particles, measured using ECLIA. C) *IL‐6* mRNA expression after 4 h of exposure to 2 µg mL^−1^ CuO/ZnO particles, measured using RT‐qPCR. D) IL‐6 protein levels after 24 h of exposure to 2 µg mL^−1^ CuO/ZnO particles, measured using ECLIA. LTA concentrations used: + for 100 ng mL^−1^ and ++ for 1000 ng mL^−1^.

**Figure 5:**
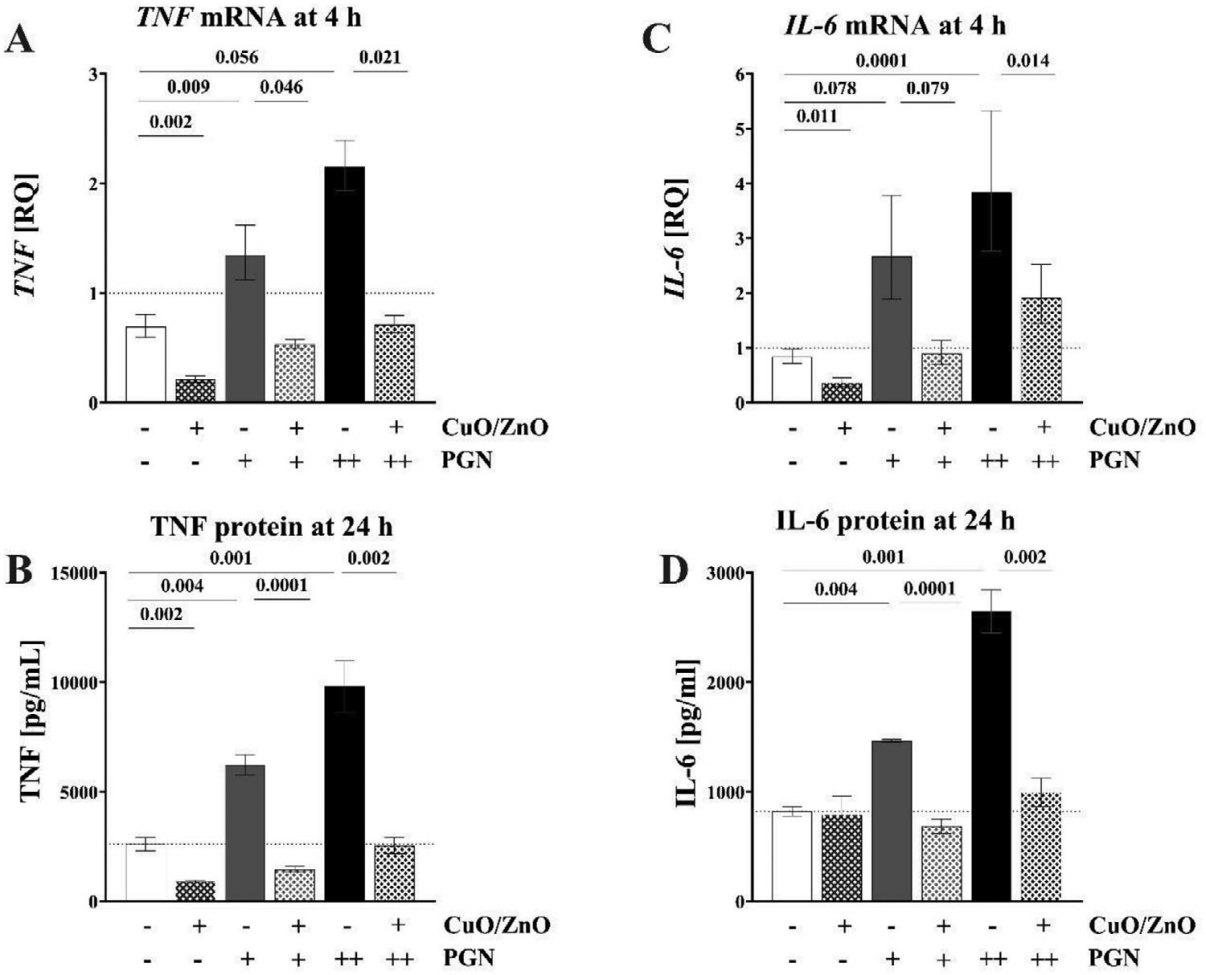
Effects of CuO/ZnO particles on THP‐1 macrophages. A) *TNF* mRNA expression after 4 h of exposure to 2 µg mL^−1^ CuO/ZnO particles, measured using RT‐qPCR. B) TNF protein levels after 24 h of exposure to 2 µg mL^−1^ CuO/ZnO particles, measured using ECLIA. C) *IL‐6* mRNA expression after 4 h of exposure to 2 µg mL^−1^ CuO/ZnO particles, measured using RT‐qPCR. D) IL‐6 protein levels after 24 h of exposure to 2 µg mL^−1^ CuO/ZnO particles, measured using ECLIA. PGN concentrations used: + for 1000 ng mL^−1^ and ++ for 3162 ng mL^−1^.

THP‐1 macrophages exposed in vitro to the bacterial components LPS (2 or 20 ng mL^−1^), LTA (100 or 1000 ng mL^−1^), or PGN (1000 or 3162 ng mL^−1^) showed a dose‐dependent increase of *TNF* and *IL‐6* mRNA expression at 4 h in comparison to the corresponding unexposed control at 4 h (**Figures** **3** **–** **5**, panels A and C; *n* = 5). This effect was more pronounced for LPS and LTA than for PGN but for all 3 PAMPs distinct. Compared to cells exposed to PAMPs alone, co‐exposure to 2 µg mL^−1^ CuO/ZnO particles alongside PAMPs resulted in significant inhibition of the expression of *TNF* mRNA and *IL‐6* mRNA levels (Figures 3, 4, 5, A and C; *n* = 5). In order to confirm a biological significance, we next investigated whether corresponding effects could also be detected for secreted TNF and IL‐6 protein levels (in the in vitro macrophage culture supernatants) after 24 h of exposure. Indeed, a dose‐dependent increase of TNF and IL‐6 protein levels following exposure to bacterial compounds (LPS, LTA, or PGN) was inhibited by 2 µg mL^−1^ CuO/ZnO particles (Figures 3, 4, 5 and B and D; *n* = 5). This inhibition of the innate immune response was more pronounced for TNF protein than for IL‐6, but in both cases distinct.

Furthermore, exposure to CuO/ZnO particles, without the presence of any PAMPs, significantly inhibited macrophage *IL‐6* mRNA and *TNF* mRNA expression at 4 h, and macrophage TNF protein secretion at 24 h, while IL‐6 protein secretion at 24 h was not affected (Figures 3–5, A, B, C, and D; *n* = 5).

### 3.4 Metal Fume Exposure and Its Impact on Antibacterial Macrophage Function and Lung Immunity

While human *short*‐term exposures to ZnO fumes have been found to increase levels of IL‐6, TNF, and IL‐8 in broncho‐alveolar lavage (BAL) fluid,^[^ ^35^, ^36^, ^38^, ^39^, ^67^, ^68^ ^]^ analysis of blood and induced sputum from long‐term welders showed no difference in inflammatory markers from unexposed controls.^[^ ^69^ ^]^ Thus, long‐term inhalation and corresponding higher doses of metal fumes may dampen the normal pro‐inflammatory response to inhaled particulate.^[^ ^69^ ^]^ This observation is in line with the fact that the incidence, severity, and death rates of bacterial pneumonia are higher among long‐term metal workers than in the general population.^[^ ^14^, ^15^, ^16^, ^17^, ^18^, ^19^, ^20^, ^21^ ^]^ For these reasons, pneumococcal vaccines are recommended for long‐term metal workers in England and Germany.^[^ ^17^, ^19^, ^21^ ^27^, ^28^, ^29^, ^31^, ^32^ ^]^ Thus far, the concrete molecular mechanisms for the dampening of lung immune responses by metal fumes have not been fully elucidated.

Lanone et al. showed that Cu‐ and Zn‐based nanoparticles were, among 24 metal nanoparticles, the most toxic for human macrophages.^[^ ^50^ ^]^ In the current study, we found a significant inhibition of innate macrophage responses to gram‐positive and gram‐negative bacterial PAMPs in the presence of occupationally relevant CuO/ZnO particles. The inhibition could be consistently shown for macrophage mRNA expression and protein secretion of two cytokines, TNF and IL‐6. Both cytokines are highly important for the immune defense against bacterial lung infections. TNF, crucial for leukocyte recruitment and activation for pathogen clearance,^[^ ^70^ ^]^ has been shown to play an essential role in the protection against gram‐positive *Streptococcus pneumoniae* infections in murine models. In mice intranasally infected with *Streptococcus pneumoniae*, TNF knockout (KO) (or pre‐treatment with a neutralizing anti‐TNF monoclonal antibody) led to higher bacterial load in the lungs, and earlier death from pneumococcal pneumonia in comparison to control wild‐type mice (or to mice without anti‐TNF pre‐treatment, respectively).^[^ ^71^, ^72^ ^]^ In addition, during pneumonia in mice caused by either gram‐negative *Escherichia coli* or *Pseudomonas aeruginosa*, TNF deficiency significantly increased bacterial burdens in the lungs 100 000‐ to 10 000 000‐fold and consequently increased mortality.^[^ ^73^ ^]^ IL‐6 activates the acute phase response and the specific immune system, e.g., Th17 cells, which are important for clearance of *Streptococcus pneumoniae* in the airways.^[^ ^74^ ^]^ After *Streptococcus pneumoniae* infection, IL‐6^−/−^ mice showed an attenuated acute‐phase protein response, higher bacterial loads in the lungs, and earlier death due to pneumococcal pneumonia compared to IL‐6^+/+^ mice.^[^ ^75^ ^]^ Both murine cytokines, TNF and IL‐6, were also reported to contribute to the clearance of *Staphylococcus aureus*.^[^ ^76^, ^77^ ^]^ Consistent with these mice model results, anti‐TNF biologics and anti‐IL‐6 biologics, therapeutically applied in humans to treat inflammatory and/or autoimmune diseases, are both independently reported to contribute to an increased risk of serious infections, including pneumonia, sepsis, and/or reactivation of tuberculosis.^[^ ^78^, ^79^, ^80^, ^81^ ^]^ The current study shows that CuO/ZnO particles inhibit the two cytokines TNF and IL‐6, both of which are essential for anti‐bacterial lung defense. This provides a potential mechanistic explanation on a molecular level for the dampening of the innate lung immune system, which may occur in long‐term metal workers. The suppression of antibacterial lung immunity by another metal, chromium, has already been reported. Exposure of rats to stainless steel welding fumes led to significant lung injury, delayed bacterial clearance, and altered immune responses. In a follow‐up study, soluble chromium in stainless steel welding fumes was identified as the primary component responsible for the described suppression of lung defense responses.^[^ ^82^, ^83^ ^]^ Future studies could further investigate whether exposure to Cu, Zn, and/or other metals (single or in combination) is immunosuppressive and whether metal exposure affects, for example, the expression of the pattern recognition receptors TLR4 (for LPS) or TLR2 (for LTA) on the surface of human macrophages.

Zn and Cu, as essential trace elements, are crucial for optimal innate immune functions. Nutritional deficiency of either metal compromises neutrophils and macrophages and leads to increased susceptibility to bacterial infection.^[^ ^84^, ^85^, ^86^, ^87^, ^88^, ^89^ ^]^ Macrophage functions can also be inhibited by excess supplementation of Cu^[^ ^90^ ^]^ or Zn.^[^ ^84^ ^]^ In contrast, moderate doses of Cu or Zn ions can be utilized by phagocytes as “contact killing” agents against intracellular bacteria, or they limit Zn availability to pathogens by sequestering it away from them, concepts known as “nutritional immunity.”^[^ ^85^, ^91^, ^92^, ^93^, ^94^, ^95^ ^]^

On the other hand, inhaled Cu and Zn may act as nutrients and enzyme cofactors for lung bacteria, thus promoting their growth.^[^ ^85^, ^94^, ^96^, ^97^ ^]^ In previous reports, it has been suggested that inhalation of another metal, iron, may lead to the release of free iron in the lung, which may act as a nutrient for pneumococci in the lung.^[^ ^98^ ^]^ This context was discussed as a reason that occupations that entail exposure to iron fumes or dust have been reported to carry an increased risk of infection.^[^ ^98^ ^]^ Moreover, a mild steel welding fume, which contained 12% iron and 3.8% manganese, has been shown, in vitro and in a mouse model, to increase the host‐expressed platelet‐activating factor receptor (PAFR) protein expression, and thereby to increase PAFR‐dependent pneumococcal adhesion and infection of lower airway cells.^[^ ^99^ ^]^

In the current in vitro cell culture study, we used 2 µg mL^−1^ CuO/ZnO particles. To establish a relationship between short‐term and/or long‐term lung exposure doses in occupational exposure scenarios, and appropriate in vitro exposure doses is not trivial. The deposition and clearance rates of inhaled nanoparticles in the respiratory system with its bifurcate tree structure are very complex to quantify, and can also vary depending on the location.^[^ ^55^, ^100^ ^]^ Furthermore, there are naturally many limitations of in vivo experiments in volunteers and patients for ethical reasons,^[^ ^100^ ^]^ which necessitates indirect research approaches for realistic dose estimations. According to the computational multiple‐path particle dosimetry (MPPD) model (www.ara.com/mppd), the pulmonary deposition of metal particles after a short‐term (6 h) exposure of healthy individuals to occupationally relevant Zn‐ and Cu‐containing welding fume at a controlled average fume concentration of 2.5 mg m^−3^ was approximately 1 mg.^[^ ^49^ ^]^ Recently, in a review dealing with modeled lung deposition and retention of welding fume particles in occupational scenarios in comparison to in vitro doses, a year of occupational exposure to an occupational exposure limit (OEL) of 5 mg m^−^^3^ was suggested to result in alveolar retention exceeding the cell dose following the highest concentration (100 µg mL^−1^) tested in vitro.^[^ ^55^ ^]^ Furthermore, real‐life occupational lung doses over both short and long‐term exposure were found comparable to cell doses exerting toxic effects on human bronchial epithelial cells in vitro.^[^ ^55^ ^]^ On the other hand, we selected with 2 µg mL^−1^ a dose with no significant toxic effects on macrophages in vitro, considering that no significant death of lung immune cells is known among welders. The non‐toxicity of 2 µg mL^−1^ Zn‐ and Cu‐containing particles is confirmed by investigations using precision‐cut lung slices (PCLS) derived from mice, rats, and humans, which demonstrated no toxicity at 0.1 and 1 µg mL^−1^ Zn‐ and Cu‐containing welding fume particle concentrations and concentration‐dependent toxicity at 10 and 100 µg mL^−1^.^[^ ^101^ ^]^ Based on these assumptions, the dose of 2 µg mL^−1^ Zn/Cu metal particles is a realistic dose, particularly for long‐term metal workers and welders. In future studies, dose‐response investigations over several time points will have to be considered. The current study paves the way for targeted investigations of the immunotoxicity of occupationally relevant individual metals or combinations thereof.

The use of existing and new chemicals and the rapid development of engineered nanomaterials (or nanoscale materials) require careful toxicity and safety screenings under environmentally relevant exposure conditions.^[^ ^102^, ^103^, ^104^, ^105^, ^106^, ^107^, ^108^ ^]^ As valuable tools for chemical hazard and risk assessments, new approach methodologies (NAMs) have emerged. The use of these in silico, in chemico, in vitro, and ex vivo methodologies aims to replace animal testing whenever possible,^[^ ^109^, ^110^, ^111^ ^]^ in accordance with the 3R principle (replacement, reduction, and refinement of animal experiments).^[^ ^112^ ^]^ NAMs are increasingly recognized and accepted for chemical risk assessment by regulatory agencies worldwide.^[^ ^113^ ^]^ In addition to chemical risk assessment, NAMs could be used as supplementary data resources for the determination of maximum workplace concentrations in the working environment.^[^ ^114^ ^]^ Upon inhalation of atmospheric metal fume particles, macrophages are an essential part of the first line of defense in the lung. As macrophages are sensitive to particle‐induced toxicity, they represent ideal targets for immunological and toxicological in vitro studies.^[^ ^115^ ^]^ The current study presents an in vitro model consisting of human THP‐1‐derived macrophages exposed to bacterial PAMPs such as LPS, LTA, or PGN in the absence or presence of xenobiotics, such as occupationally relevant metal (nano‐)particles. Methodically, this pathogen‐macrophage infection model enables future investigations of potential immunosuppressive effects of inhaled xenobiotics at workplaces. Our non‐animal model may also extend or complement the macrophage cytokine release assay^[^ ^116^ ^]^ or other existing NAMs.^[^ ^112^, ^117^, ^118^ ^]^

## 4 Conclusions

The results of the current study show that macrophage TNF and IL‐6 mRNA and protein responses to the PAMPs LPS, LTA, or PGN are significantly inhibited in the presence of 2 µg/mL welding‐fume derived CuO/ZnO particles. Thereby, innate immunity to bacterial pneumonia may be weakened because human macrophages are part of the lung's first‐line immune defense,^[^ ^51^, ^52^, ^53^, ^54^ ^]^ and the cytokines TNF and IL‐6, which they release (when uninhibited), are essential for defense against bacterial lung pathogens, such as *Streptococcus pneumoniae*.^[^ ^71^, ^72^, ^73^, ^74^, ^75^, ^76^, ^78^, ^79^, ^80^, ^81^, ^119^, ^120^ ^]^ Since we observed similar results for bacterial PAMPs from Gram‐positive bacteria (LTA, PGN) and Gram‐negative bacteria (LPS, PGN), these results may apply to a broad spectrum of bacteria involved in human pneumonia. The interference of CuO/ZnO particles with the innate immune system's ability to combat lung pathogens provides potential mechanistic explanations for the increased susceptibility to severe or even lethal pneumonia observed in some metal workers.^[^ ^14^, ^15^, ^16^, ^17^, ^18^, ^19^, ^20^, ^21^ ^]^ The elevated CuO/ZnO doses for the immuno‐toxicological effects may mainly occur in the lungs of metal workers after occupational long‐term metal fume exposure, which is indeed held responsible for increased severe pneumoniae and even death rates among long‐term metal workers.^[^ ^16^, ^21^ ^]^ Studies by the group of Antonini also showed that chromium in stainless steel welding fumes led to significant lung injury, delayed bacterial clearance, and altered immune responses in mice models.^[^ ^82^, ^83^ ^]^ Future research will need to explore, possibly by including our in vitro model approach and already existing NAMs, whether the suppression of anti‐bacterial lung immunity is also applicable to other metal fume components.

To avoid the dampening of the human lung immune responses to bacterial lung infections, and possibly any unintended nutritional effects^[^ ^15^, ^18^, ^85^, ^94^, ^96^, ^97^, ^98^ ^]^ on pathogenic lung bacteria, by CuO/ZnO fumes at working places, our results emphasize the need for careful consideration of preventive occupational health guidelines, including suction devices and respirators at the working place, and compliance to occupational exposure limits for ZnO and CuO. Other research groups even recommend a re‐evaluation of occupational exposure limits for ZnO.^[^ ^45^, ^46^ ^]^ Moreover, our results emphasize that the current vaccine recommendations for long‐term metal workers are helpful in protecting them from the adverse health impacts of metal fume exposures.

## Conflict of Interest

The authors declare no conflict of interest.

## Data Availability

The data that support the findings of this study are available from the corresponding author upon reasonable request.
